# Advances and prospects of genomic-assisted breeding in roots, tubers, and banana crops

**DOI:** 10.3389/fpls.2026.1744272

**Published:** 2026-03-04

**Authors:** Paterne A. Agre, Brigitte Uwimana, Edwige Gaby Nkouaya Mbanjo, Morag Ferguson, Ranjana Bhattacharjee, Moses Nyine, Vishnuvardhan Banda, Prasad Peteti, Sikirou Mouritala, Kayondo S. Ismail, Delphine Amah, Yao Kolombia, Michael Batte, Jaindra N. Tripathi, Valentine Nakato, Trushar Shah, Rony Swennen, Asrat Asfaw, Rabbi Y. Ismail, Leena Tripathi, Hapson Mushoriwa

**Affiliations:** 1 International Institute of Tropical Agriculture, Ibadan, Oyo, Nigeria; 2 International Institute of Tropical Agriculture, Namulonge, Uganda; 3 International Institute of Tropical Agriculture, Nairobi, Kenya; 4 International Institute of Tropical Agriculture, Kinshasa, Democratic Republic of Congo; 5 International Institute of Tropical Agriculture, Dar Es Salaam, Tanzania

**Keywords:** accelerated breeding, advanced breeding, breeding optimization, genomic-assisted breeding, RTB

## Abstract

Roots, Tubers, and Bananas (RTB), including banana and plantain, cassava, yam, sweetpotato, and potato crops, share several defining features that set them apart from cereals and legumes. They are essential for food and nutritional security for hundreds of millions of people, especially in developing countries. Despite their significance, RTB crop breeding has lagged due to the complexity of genetics and the use of vegetative propagation. Recent advancements in genomics-assisted breeding (GAB) offer opportunities to accelerate genetic gains. This review provides an updated synthesis of genomic tools and associated strategies applied in RTB breeding, with a focus on banana and plantain, cassava, and yam. It spans the recent development and application of genomic tools, from diversity studies and trait discovery to marker-assisted selection, genomic selection, pan-genomics, and gene editing. It also highlights the efforts to modernize RTB breeding through program optimization and digital tool integration. The review concludes by suggesting future directions for sustainable impact.

## 1 Introduction

Roots, Tubers and Bananas (RTB) is a term coined to represent banana and plantain (*Musa* spp.), cassava (*Manihot esculenta*), potato (*Solanum* tuberosum), sweetpotato (*Ipomoea batatas* Lam.), and yam (*Dioscorea* spp.). Mostly grown in the tropical and subtropical regions, RTB crops are the pillar of food and nutritional security in developing countries, and more importantly in sub-Saharan Africa, contributing to calorie intake ranging from 25% to 57% (Thiele and Friedmann, 2020). They are an indispensable source of livelihood for over two billion people. In some countries, they are predominant over cereal crops owing to their monetary value and capacity to bridge the poverty gap (Wiebe et al., 2021). The production of most RTB crops has steadily increased since 1973, with an average growth of 233% by 2023, the highest increase coming from yam (605%), while sweet potato production, on the other hand, has dropped by 49% (Food and Agriculture Organization of the United Nations (FAO), 2023). A further increase in production is expected over the next 25 years, especially in sub-Saharan Africa, where RTB crops are forecast to surpass other staple crops, although this increase will still fall short of the growing demand (Petsakos et al., 2019; Wiebe et al., 2021). RTB crops are primarily subsistence commodities used as food and animal feeds, with the exception of cassava, which is an emerging source of industrial processing products such as edible starch, ethanol, and gluten-free baking flour (Sengar, 2022). Both cassava and yams have the capacity to withstand harsh growing conditions such as poor soils and drought, and their long-term underground root/tuber storage makes them essential crops for poverty reduction and economic development in sub-Saharan Africa (Burns et al., 2010; Amelework et al., 2021). Bananas and plantains are perennial crops that provide food year-round, serving as an agricultural backbone and a vital fallback for smallholder farmers during extremely dry seasons when annual crops fail (Scott, 2021).

Genetic improvement of RTB crops is challenging due to their high genetic complexity. Polyploidy and multiple species and subspecies of bananas, plantains, and yams complicate genetic analyses and inheritance predictions. High heterozygosity not only masks deleterious alleles in all these crops (Ramu et al., 2017), but also makes it harder to fix desired alleles across generations (Friedmann et al., 2018). The rare flowering in the case of yam and asynchronous flowering in cassava, the poor seed set and low germination rate observed in all these crops, coupled with a dioecious nature in the case of yam, limit genetic recombination, genetic diversity and slow down breeding progress (Ibrahim et al., 2020; Mondo et al., 2020). Furthermore, the crops face various biotic and abiotic constraints that greatly reduce their yield (Ploetz et al., 2015; McCallum et al., 2017; Uwimana et al., 2021b; Asfaw et al., 2024b; Tariq et al., 2024). Propagated vegetatively using suckers, stem cuttings, tubers/mini-setts, or vines, RTB planting materials are not easily conserved, multiplied, or transported. This mode of propagation also increases the risk of disease and pest transmission through the use of unhealthy planting material, which is common in the poorly developed seed systems of the RTB crops (Andrade-Piedra et al., 2016). The development of new varieties that combine resistance, yield, and consumer quality traits through conventional breeding can take up to two decades, as is the case with bananas and plantains (Tenkouano et al., 2019). These constraints call for a search for alternative breeding strategies and tools to shorten breeding cycles, increase genetic gain to close the yield gaps, and meet the consumption needs of an ever-growing population.

The recent technological innovations, especially advancements in genomic technologies and their reduced cost, offer new opportunities for RTB crops. Integration of genomic information and molecular tools has shown their prowess in numerous other crops, complementing the conventional approach, improving breeding accuracy and efficiency, and resulting in the release of numerous improved varieties with desired characteristics (Mangal et al., 2024). CGIAR research program (CRP) on RTB was launched in 2012 to accelerate the implementation of genetic innovation in RTB crops and address the challenges in breeding faced by these crops, and expedite the development of improved varieties that meet end-users’ preferences (Almekinders et al., 2019).

With a focus on banana and plantain, yam and cassava, the three RTB breeding programs at the International Institute of Tropical Agriculture (IITA), this paper aims to: (1) review the genomic resources available for different RTB crops; (2) assess how these resources have contributed to unlocking the value and use of the potential genetic resources; (3) review the use and impact of genomics throughout the development process; (4) evaluate different tools and strategies used or explored to expedite the genetic improvement of these vegetatively propagated crops.

## 2 Genomic resources and technologies in RTB crops

The last two decades have come with a tremendous boost in the development of reference genomes of the RTB crops, previously regarded as orphan crops for genomic research. Due to their high levels of heterozygosity, the initial reference genomes were constructed using double-haploid genotypes to simplify the assembly process (D’Hont et al., 2012; Wang et al., 2019). Advancements in long-read sequencing technologies have facilitated the analysis of highly heterozygous, polyploid, and complex genomes, leading to both the improvement of original RTB reference genomes and the development of new, haplotype-resolved references that are more relevant for trait discovery and breeding purposes (Tamiru et al., 2017; Belser et al., 2021; Landi et al., 2023; Xu et al., 2023; Chen et al., 2024). Combined with low-cost, sequencing-based genotyping platforms such as Diversity Array Technology sequencing (DArTSeq), Genotyping By Sequencing (GBS), and Single Nucleotide Polymorphic (SNP) array technologies, the high-quality reference genomes have enabled the generation of large genome-wide marker datasets for diversity studies (Ferguson et al., 2019; Agre et al., 2023; Akech et al., 2024) and association of markers with key traits in all RTB crops (Nyine et al., 2019; Agre et al., 2021b; Uwimana et al., 2021a; Rabbi et al., 2022). High-quality reference genomes have greatly improved the accuracy of gene annotations, leading to the identification of candidate genes associated with key traits such as disease resistance, stress tolerance, and yield. Based on these annotations, gene expression analyses were performed to understand when and where specific genes are active, providing further insight into their roles in trait development and response to environmental conditions (Olayide et al., 2020; Liu et al., 2021; Backiyarani et al., 2022).

Reference genomes in crops have facilitated the construction of pangenomes, which represent the full set of genes across various cultivars and species. These include core genes shared by genomes of all genotypes included in the panel (typically linked to essential functions), useful genes found in some but not all genomes (often related to adaptation, disease resistance, or quality traits), and unique genes present only in specific wild relatives or landraces, offering valuable resources for novel trait discovery and pre-breeding efforts. While some pangenomes have been constructed for RTB crops, specifically for banana and cassava (Rijzaani et al., 2022; Xia et al., 2025), additional efforts are needed to incorporate recently developed reference genomes and diverse genotypes from different locations/regions, enabling the integration of evolutionary studies with breeding applications.

In banana/plantain and cassava, comparative pangenomic and synteny studies have uncovered extensive chromosomal structural rearrangements that have contributed to the process of speciation and identification of underlying key traits such as cross-compatibility, seed set, disease resistance, abiotic stress tolerance, and tuber or root quality. These studies have also shed light on the ancestral composition of domesticated genotypes, providing valuable insights into the evolutionary history and domestication pathways (Martin et al., 2020a, Martin et al., 2020b; Lyons et al., 2022; Martin et al., 2025). These findings are instrumental in linking evolutionary dynamics with breeding strategies aimed at improving the performance and resilience of RTB crops.

## 3 Implementation of marker technologies

### 3.1 Genomic resources to unlock germplasm value and exploit genetic resources

Molecular markers are vital tools for effective pre-breeding and conservation. Different markers systems, including Simple Sequence Repeats (SSRs), Random Amplified Polymorphic DNA (RAPD), and Amplified Fragment Length Polymorphism (AFLP) have been used for molecular characterization of RTB crops (Pillay et al., 2001; Fregene et al., 2003; Egesi et al., 2007; Christelová et al., 2016). More recently, Single Nucleotide Polymorphisms (SNPs) have gained popularity owing to their high abundance, cost-effectiveness, and amenability to high-throughput genotyping (Mammadov et al., 2012). Molecular markers have been used in RTB breeding programs to depict genetic relationship among genotypes, for parentage verification, and better understanding of population structure, which has enhanced core collection development and served to optimize the breeding diversity (Bredeson et al., 2016; Agre et al., 2021a, Agre et al., 2023; Santos et al., 2023; Akech et al., 2024). Molecular markers also support true-to-type dissemination and have been used to evaluate the impact of cassava improvement (Rabbi et al., 2015). They have also been shown to be trustworthy for genotype authentication and duplicate identification (Agre et al., 2021a; Carvajal-Yepes et al., 2024; Mbanjo et al., 2024). The use of trait-associated markers has helped breeders identify useful alleles present in wild relatives and landraces and their introgression into elite germplasm. Genomic information has also shed light on the domestication processes and the role of introgression in modern breeding (Wolfe et al., 2016; Martin et al., 2020b, Martin et al., 2023). Overall, the progressive application of molecular markers has significantly advanced genetic characterization, conservation, and breeding efficiency in RTB crops. The transition from earlier marker systems to high-throughput SNP genotyping has deepened understanding of genetic diversity and population structure, facilitated more precise parent selection and germplasm management, and strengthened the molecular basis for trait improvement and domestication studies.

### 3.2 Trait discovery and deployment

**Figure 1:**
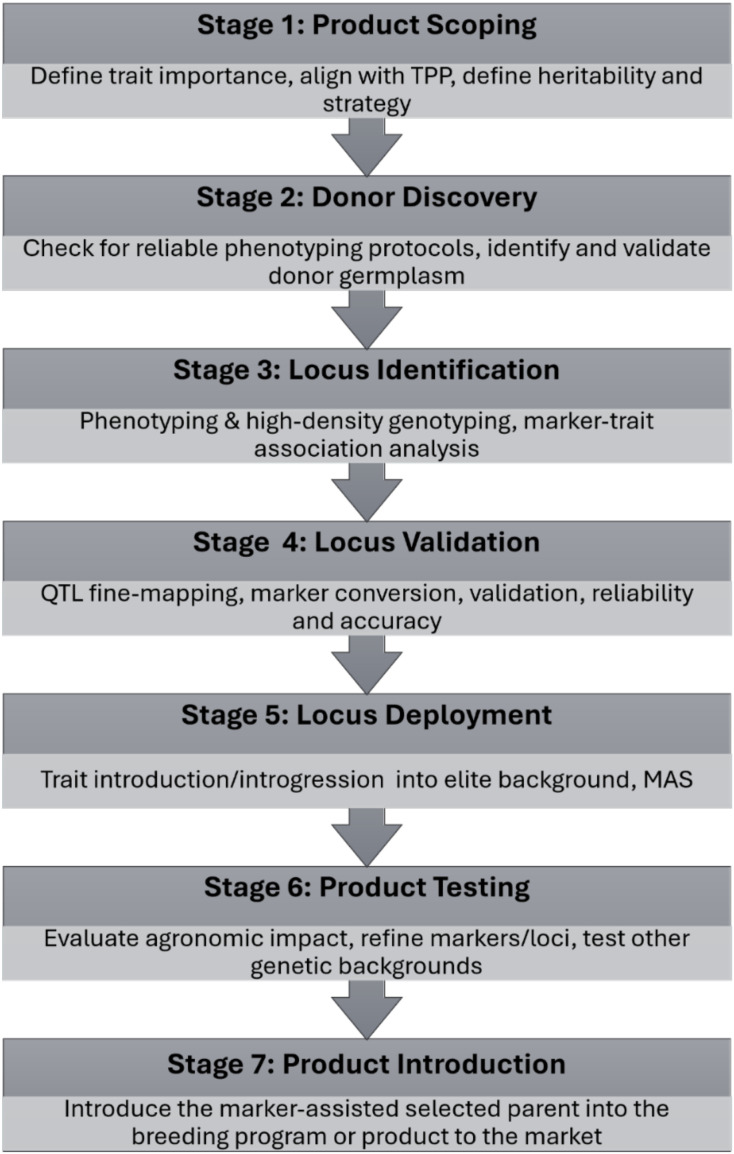
A graphic representation of the trait discovery stage gates, from product scoping to the introduction.

Trait Discovery and Deployment (TD&D) is a core strategy in genomics-assisted breeding, aimed at identifying beneficial genetic variation and efficiently introducing it into elite germplasm without compromising population integrity. The TD&D process involves seven key stages (Platten et al., 2025) as presented in **Figure 1**. TD&D is challenged in clonally propagated RTB crops due to somatic mutations, low recombination, and chimerism (Ramu et al., 2017), but modern tools and targeted strategies are increasingly overcoming these barriers. Briefly, in Stage 1 (product scoping), the breeding team defines the trait’s importance based on the target product profile (TPP), the trait heritability, and decides on a suitable strategy, typically QTL mapping or GWAS, depending on population structure and available resources. Stage 2 (donor discovery) entails identifying and validating donor germplasm from elite lines, landraces, or wild relatives. Techniques such as allele mining, mutant screening, and gene editing can be used. Stage 3 (locus identification) relies on high-density genotyping and precise phenotyping to identify significant QTL or markers. In Stage 4 (locus validation), if deemed necessary, the identified QTL can be fine-mapped. The identified markers (often SNPs) are converted to high-fidelity, repeatable, low-density markers, often Kompetitive Allele Specific PCR (KASP). The KASP-SNP markers are tested for their trait prediction accuracy and reliability in different genetic backgrounds. Stage 5 (locus deployment) involves the introduction of the trait through marker-assisted selection (MAS), often followed by introgression to augment the value of the elite parent. In Stage 6 (Product testing), the economic value and agronomic impact of the product carrying a new trait are evaluated. Marker or locus refining is carried out if judged necessary, preferably through transcriptomics and multi-omics technologies. Stage 7 (product introduction), the products (elite donors, marker systems) are introduced into the breeding programs, or the first marker-selected product is introduced to the market. At this level, MAS becomes routine as the marker/locus and marker toolkit integrate into the breeding pipeline for broader deployment.

#### 3.2.1 Trait discovery

In recent years, RTB breeding programs have invested in molecular trait discovery, which has resulted in the association of molecular markers with key traits following the TPPs as summarized in **Table 1**. **Table 1** gives a comprehensive overview of the progress made by the banana and plantain, cassava, and yam breeding programs with respect to trait discovery. (RTB stage discovery).

**Table 1:** Marker-trait association studies in RTB crops in the last ten years.

Crop	Traits	Reference
Bananas and plantains	Seedlessness and parthenocarpy	Sardos et al. (2016)
	Banana bunch weight and yield-related traits	Nyine et al. (2019)
	Resistance to Fusarium wilt (*Fusarium oxysporum* f. sp. *Cubense*) race 1 and tropical race 4	Ahmad et al. (2020)
	Resistance to banana weevils (*Cosmopolites sordidus* (Germar))	Uwimana et al. (2021a)
	Resistance to banana Fusarium wilt (*Fusarium oxysporum* f. sp. *Cubense*) subtropical race 4	Chen et al. (2023a) and Chen et al. (2023b)
	Resistance to banana bacterial wilt (*Xanthomonas vasicola pv. musacearum*)	Uwimana et al. (2024)
	Resistance to black leaf streak (*Pseudocercospora fijiensis*)	Carreel et al. (2024)
	Morphology, fruit quality and yield traits	Osorio-Guarin et al. (2024)
Cassava	Starch pasting viscosity	Thanyasiriwat et al. (2014)
	Cyanogenic, glucose and dry matter content	Balyejusa Kizito et al. (2007); Whankaew et al. (2011); Ogbonna et al. (2021); Ogbonna et al. (2020)
	Yield	OkogBenin and Fregene (2002); Liu et al. (2025);
	Resistance to cassava bacterial blight (*Xanthomonas axonopodis* pv. manihotis)	Jorge et al. (2001)
	Agronomic, Diseases and Pests	Rabbi et al. (2022); Nzuki et al. (2017); Nandudu et al. (2023); Nandudu et al. (2024); Wolfe et al. (2016); Rabbi et al. (2014); Kayondo et al. (2018); Hohenfeld et al. (2022); Ospina et al. (2024); Garcia-Oliveira et al. (2020); Ezenwaka et al. (2018); Baguma et al. (2024); Rabbi et al. (2022)
	Tuber quality (dry matter; gari/eba yield & quality (peel loss, swelling, colour, texture)	Aghogho et al. (2024); (Uchendu et al., 2021); Santos et al. (2022); Rabbi et al. (2022); Rabbi et al. (2017)
Yam	Resistance to anthracnose (*Colletotrichum gloeosporioides*)	Mignouna et al. (2002); Bhattacharjee et al. (2018); Agre et al. (2022)
	Tuber quality traits	Ehounou et al. (2022); Asfaw et al. (2024a); Arnau et al. (2024); (Gatarira et al., 2020)
	Yield traits and resistance to yam mosaic virus	Agre et al. (2021b); Adejumobi et al. (2023); Adjei et al. (2024)
	Flower sex determination and cross compatibility	Mondo et al. (2021)

#### 3.2.2 Trait deployment

For the trait linked markers to be useful in breeding, they must be successfully converted to robust, repeatable, and low-cost assays, such as KASP markers to be used in MAS. Most of the QTL identified and/or published are yet be used in RTB breeding. While few have been successfully deployed in the breeding program, the accuracy and transferability is still low for many traits due to several factors affecting the QTL stability. The choice of breeding methodology whether MAS of specific alleles, marker-assisted recurrent selection (MARS), or Genomic Selection (GS), is governed by the trait’s genetic architecture. MAS is ideal for major-effect, Mendelian loci, while GS is more effective for polygenic, quantitative traits (Meuwissen et al., 2001; Desta and Ortiz, 2014).

MAS uses markers tightly linked to traits of interest to indirectly select for genotypes that carry beneficial alleles and eliminate undesirable genotypes early in the breeding process. The numerous trait-linked markers available for the RTB crops (https://excellenceinbreeding.org/module3/kasp) are useful tools to speed up the early identification of high-value genotypes and minimize the confounding effect of the environment. Successful stories demonstrating the effective use of markers to select target QTL in RTB crops are limited. MAS has mostly been used for monogenic traits in foreground selection. A successful use of molecular markers has been reported in cassava for resistance to cassava mosaic disease (Ceballos et al., 2015). Molecular markers linked to a major dominant gene, CMD2, associated with cassava mosaic disease have been used to evaluate the offspring from crosses, and identify which ones carry the resistant gene and those resistant lines are shared with various breeding programs (Blair et al., 2007; Parkes et al., 2015). More recently, the EMBRAPA-led GWAS for hydrogen cyanide (HCN) content in cassava (Ogbonna et al., 2021) identified major-effect loci on chromosomes 14 and 16, with diagnostic SNP markers now validated in African and Brazilian germplasm, demonstrating the feasibility of marker deployment for root quality traits. Recently, several proof-of-concept studies have been conducted in various RTB crops to validate the utility of trait-associated markers. An allele-specific molecular marker was used to identify genotypes that carry mutant granule-bound starch synthetase (GBSSI) for waxy starch in cassava (Aiemnaka et al., 2012; Carmo et al., 2015). Likewise, markers from a high-effect single QTL are proving useful to eliminate banana hybrids without pulp at the earliest evaluation stage (Nyine et al., 2019). Additionally, the predictive ability of a number of cassava trait-linked markers has been evaluated (Esuma et al., 2022; Ige et al., 2022; Mbanjo et al., 2024; Kanaabi et al., 2024). More recently, an informative SNP marker associated with Fusarium oxysporum f. sp. cubense (Foc) Tropical Race 4 (TR4) was used to assess the presence of a resistance allele in the banana breeding material in Africa and a collection of banana accessions in Brazil (Chen et al., 2023b; Ferreira et al., 2024). Similarly, the potential of molecular markers for early identification of plant sex in yams was demonstrated (Agre et al., 2020).

The mainstreaming of the developed resources in breeding programs is, nevertheless, hindered by persistent challenges. Indeed, trait-associated markers are ineffective in some genetic backgrounds or show considerable levels of false positives and/or false negatives. Additional loci, not yet captured and contributing to the traits, might be limiting their predictive accuracy in diverse germplasm, false positives/negatives, and incomplete capture of genetic variance. MARS is a more appropriate approach for combining several genes or QTLs simultaneously. MARS is achieved by crossing parental lines with complementary desirable alleles/genes identified from each QTL profile and selecting the desired recombinant genotypes with optimal complement QTL and intercrossing them (Ceballos et al., 2015; Gokidi et al., 2016). Early marker-assisted selection (MAS) implemented in a breeding program will aid in large-effect QTL selection, lowering the number of genotypes that need to be phenotyped in subsequent generations. By using markers, the period required for trait introgression is shortened. In a few generations, favourable alleles could be fixed, and the frequency of favourable alleles in the breeding population will rise rapidly. This limitation is exacerbated in RTB crops by high heterozygosity and variable ploidy levels, where markers discovered in diploid populations may not validate consistently in polyploid or clonal backgrounds.

### 3.3 Genomic selection

Another well-proven approach in GAB is genomic selection (GS), particularly valuable for complex traits. GS predicts the breeding value of individuals using genome-wide markers, eliminating the need for repeated phenotyping. formalized by Meuwissen et al. (2001). GS builds upon traditional statistical frameworks like the infinitesimal model (Fisher, 1919) and Best Linear Unbiased Predictions (Henderson, 1985). GS is routinely implemented in cassava to predict key traits such as resistance to cassava mosaic disease (CMD), cassava brown streak disease (CBSD), and dry matter content, with accuracies ranging from 0.52 to 0.68 (Wolfe et al., 2017; Esuma et al., 2021). Prospective parents are selected based on their genetic values determined using marker information. There is an ongoing effort to integrate GS in all RTB crops. In yams, the potential of GS to predict traits like dry matter and tuber color has been demonstrated with a prediction accuracy up to 0.97 (Agre et al., 2023; Norman et al., 2023). Additionally, Nyine et al. (2018) demonstrated GS prediction accuracies ranging from 0.45 to 0.75 for yield and agronomic traits in banana, predominantly in triploid genotypes. In bananas and plantains, GS holds even greater promise in diploid (2*x*) and tetraploid (4*x*) parental improvement schemes, where recurrent selection is feasible and can be implemented more effectively. By contrast, in yam and banana, GS is presently used mainly for parental selection and early-stage advancement, and no released varieties have yet been selected exclusively based on genomic estimated breeding values.

Despite notable advancements in model evaluation, GS remains underused in RTB breeding due to resource limitations and a lack of capacity in high-throughput. In recent years, many RTB breeding programs have transitioned from genotyping-by-sequencing (GBS) to DArTseq. Both approaches rely on reduced-representation sequencing, but the discontinuation of GBS services led to DArTseq becoming the routine platform for genomic selection in RTBs. A further bottleneck is bioinformatics: sequencing-based genotyping generates large volumes of raw data that require specialized pipelines for SNP calling, imputation, and quality control. Limited computational infrastructure and trained personnel often slow routine GS analyses, highlighting the need for investment in shared cloud-based platforms, standardized pipelines, and capacity building across breeding programs. To fully exploit its potential, investments in bioinformatics, high-throughput precision phenotyping infrastructure, and AI integration are needed, alongside harmonized pipelines and cross-crop learning (Ceballos et al., 2015). Another GS approach deployed in RTB is the use of Genomic Prediction of Cross Performance (GPCP) and Optimal Contribution Selection (OCS), which are revolutionizing parental selection strategies (Agre et al., 2023). Both approaches, which integrate genomic information, aims to increase genetic gain but with different focus. GPCP enables to identify crosses likely to produce progenies with desired trait, while OCS provides an optimization framework that balances genetic gain with genetic diversity (Clark et al., 2012). In cassava and yam, these strategies have been adopted to design crosses that strategically accumulate favorable alleles while maintaining additive and dominance effects in the population (Wolfe et al., 2017). A complementary innovation in GS implementation is Sparse testing, which strategically allocates phenotyping resources to only a subset of genotypes across environments, while the remaining genotypes are evaluated solely through genomic prediction. This approach reduces the cost and labor intensity of multi-environment testing, while maintaining or even improving prediction accuracy (Jarquin et al., 2020). Sparse testing has been successfully applied in maize and wheat and is now being piloted in cassava breeding programs (Lubanga et al., 2025), where large early-generation populations and limited resources make it especially relevant. By leveraging genomic relationships and genotype-by-environment models, Sparse testing allows breeders to maximize information gain while minimizing experimental effort, ultimately enhancing the efficiency of GS-driven selection pipelines. Sparse testing has been successfully applied in maize and wheat and is increasingly being considered in RTB breeding, where large population sizes (early generation trials) and resources are limited, thus making it especially relevant. This is particularly important for RTB crops, where slow vegetative propagation and bulky planting materials limit the number of locations to which clones can be distributed during early testing phases.

### 3.4 Genome editing

In the last decade, genome editing has revolutionized crop improvement by enabling precise modifications to target genes. Among the available tools, CRISPR/Cas systems have emerged as the most widely adopted due to their simplicity, flexibility, and efficiency. CRISPR/Cas is increasingly being used in RTB crops, especially to improve disease resistance, starch quality, plant architecture, and abiotic stress tolerance. **Table 2** summarises recent advancements in genome editing of RTB crops, banana, plantain, cassava, and yam.

**Table 2:** Summary of recent advances in genome editing of RTB crops mainly banana, cassava, and yam.

Crops	Trait	Editing system	Explant	Target	References
Banana	Albino phenotype	CRISPR-Cas9	Embryogenic cells	*MusaPDS*	Kaur et al., 2018; Naim et al., 2018; Ntui et al., 2020
	Increase β-carotene	CRISPR-Cas9	Embryogenic cells	*Musa lycopene epsilon-cyclase gene*	Kaur et al., 2020
	Shorter height	CRISPR-Cas9	Embryogenic cells	*gibberellin 20ox2* (*MaGA20ox2*) gene	Shao et al., 2020
	Bacterial disease resistance	CRISPR-Cas9	Embryogenic cells	*MusaDMR6* gene	Tripathi et al., 2019
	Delayed ripening	CRISPR-Cas9	Embryogenic cells	*MaACO1* gene	Hu et al., 2021
	Albino phenotype	Cas-Clover	Embryogenic cells	*MusaPDS*	Tripathi et al., 2023
	Bacterial disease resistance	CRISPR-Cas9	Embryogenic cells	*MusaPUB22/23*	Tripathi et al., 2025
Plantain	Banana Streak Virus resistance	CRISPR-Cas9	Embryogenic cells	*Endogenous Banana Streak Virus in the B genome of banana*	Tripathi et al., 2019
	Albino phenotype	CRISPR-Cas9	Embryogenic cells	*MusaPDS*	Ntui et al., 2020
	Disease resistance	CRISPR-Cas9	Embryogenic cells	*MusaENOD*3 genes	Ntui et al., 2024
Cassava	Starch content	CRISPR-Cas9	Friable calli	*Me*PTST1 or MeGBSS)	Bull et al., 2018
	Virus resistance cassava	CRISPR-Cas9	Friable calli	eIF4E isoforms nCBP-1, nCBP-2	Gomez et al., 2019
	Albino phenotype	CRISPR-Cas9	Friable calli	*MePDS*	Odipio et al., 2017
	Cyanogen free cassava	CRISPR-Cas9	Immature leaf	*MeCYP79D1*	Juma et al., 2022
	Bacterial blight resistance	Zinc figure nuclease/epigenetic methylation	Friable calli	MeSweet10a	Veley et al., 2021
	Cyanogen free cassava	CRISPR-Cas9	Friable calli	Cytochrome P450 genes *CYP79D1* and *CYP79D2*	Gomez et al., 2023
	Increasing amylose contents	CRISPR-Cas9	Friable calli	Soluble starch synthase gene (MeSSIII-1)	Lu et al., 2025
Yam	Albino phenotype	CRISPR/Cas9	Axillary buds	*DrPDS*	Syombua et al., 2025

IITA has established a robust genome editing platform for a full pathway encompassing bioinformatics, editing reagent design, generation of edited lines, molecular chacraterization and phenotyping for RTB crops. Significant progress has been made using CRISPR/Cas9-based genome editing to develop disease-resistant banana varieties by precisely editing endogenous susceptibility genes. For example, disruption of susceptibility genes such as *MusaDMR6*, *MusaENODL3*, and *MusaPUB22/23*, which are typically upregulated during pathogen infection, has yielded enhanced resistance to Banana Xanthomonas wilt (BXW) disease without adversely affecting plant growth (Tripathi et al., 2019; Ntui et al., 2024; Tripathi et al., 2025). Editing in plantain, targeting mutations within the integrated viral genome, successfully inactivated the endogenous Banana streak virus (eBSV) (Tripathi et al., 2019). Approximately 75% of the edited plants showed no BSV symptoms under greenhouse conditions, potentially enabling wider use of B-genome germplasm in global breeding programs.

Furthermore, genome editing has been applied to improve nutritional traits, such as increasing β-carotene levels in the Cavendish cultivar (Kaur et al., 2020). It has also been used to shorten the plant height by editing MaGA20ox2 (Shao et al., 2020) and delay ripening processes by reducing endogenous ethylene production through editing of the aminocyclopropane-1-carboxylase oxidase (MaACO1) gene (Hu et al., 2021). These examples demonstrate the multifaceted potential of genome editing to enhance banana quality, resilience, and productivity.

In cassava, CRISPR/Cas9 has been successfully applied to edit genes involved in various traits, leading to significant advancements in crop improvement. Gene editing in cassava was first optimized by targeting the *PDS* gene in the model cultivar TMS60444, paving the way for subsequent optimization in other cultivars (Odipio et al., 2017). Subsequent applications targeted genes regulating starch biosynthesis resulting in modified amylose content and altered starch functionality (Lu et al., 2025; Bull et al., 2018). To enhance disease resistance in cassava, early genome editing efforts targeted the blocking of the elFL4 gene isoform, resulting in edited events with reduced CBSD symptoms (Gomez et al., 2019). Subsequent studies focused on developing resistance to bacterial wilt by knocking out the MeSWEET10a disease susceptibility gene, which disrupted promoter methylation and bacterial TAL-effector binding (Veley et al., 2021). More recently, efforts have been directed toward improving food safety through the knockout of the CYP79D1 and CYP79D1 genes, producing safer cassava varieties with lower cyanide levels (Juma et al., 2022; Gomez et al., 2023).

In yam, genome editing has been demonstrated by targeting the visual marker gene *DrPDS* (Syombua et al., 2025). Following the optimization of a friable embryogenic callus-based regeneration system in yam (Syombua et al., 2025), yam can now be efficiently edited, enabling the crop to target for traits such as virus resistance and plant architecture.

Despite its potential, several barriers hinder the widespread application of genome editing in RTB crops. One of them is the development of efficient, genotype-independent transformation and regeneration systems, coupled with the recalcitrant tissue culture responses. The polyploidy and heterozygosity in some RTB crops also complicate editing of all alleles, often leading to mosaicism. Moreover, delivery methods such as Agrobacterium-mediated transformation and particle bombardment often suffer from low efficiency and can induce somaclonal variation (Divya et al., 2023). Nevertheless, the genome editing system of banana established at IITA using embryogenic cell suspension targeting the Phytoene desaturase (PDS) gene as a visual marker showed extremely high efficiency of editing with targeted mutations in all three alleles (Ntui et al., 2020).

The regulatory landscape is another critical obstacle. The classification and regulation of genome-edited crops differ widely among countries. Many African and European countries continue to apply process-based frameworks that equate genome editing with traditional genetic modification, whereas countries like the United States and Argentina have a product-based regulatory system (Lassoued et al., 2018). The adoption and commercialisation of genome-edited RTB crops are hampered by this discrepancy. Public perception and lack of awareness also influence the regulatory climate and the political will to advance genome editing. Public engagement, transparent risk assessments, and harmonisation of international policies are urgently needed to facilitate the deployment of genome-edited varieties.

The regulatory landscape for genome-edited crops varies widely across regions, influencing their adoption and commercialization (Tripathi et al., 2024). While countries such as the United States and Argentina apply product-based regulations that assess the safety of the final product rather than the editing process, allowing several CRISPR-derived crops to reach the market, European nations still follow process-based frameworks that classify genome editing alongside traditional GMOs (Lassoued et al., 2018). However, regulatory reforms are emerging, particularly in Africa, where nations like Kenya, Nigeria, Ghana, Malawi, and Ethiopia now distinguish genome-edited crops from GMOs through case-by-case assessments. Kenya has led these efforts, approving gene-edited maize and sorghum as non-GMOs, while others are developing supportive policies. Similar progress is observed in Latin America, Japan, and Australia, where regulation focuses on the absence of foreign DNA and equivalence to conventionally bred crops. Despite these advancements, challenges persist in harmonizing biosafety frameworks across regions. Strengthening science-based, risk-proportionate policies, enhancing public awareness, and promoting regional and international alignment, as advocated by organizations such as AUDA-NEPAD, are essential to enable safe commercialization and cross-border trade of genome-edited crops (Akinbo et al., 2025) Nigeria and Kenya have already approved regulatory guidelines for genome-edited crops, whereas Ghana and several other African countries are currently finalizing or piloting similar frameworks.

The public perception and acceptance of genome editing are crucial for its successful implementation. For African nations to leverage CRISPR/Cas and other NGTs to address food security, climate resilience, and hidden hunger, they must strengthen biosafety frameworks, increase public awareness, and promote regional cooperation.

### 3.5 Lessons learned from MAS in RTB crops

#### 3.5.1 Preserving MAS-derived traits through vegetative reproduction

A unique advantage of RTB crops is that once a desirable allele is fixed in a selected genotype, it is faithfully maintained across generations without segregation. This stability ensures that genetic gains achieved through MAS are preserved in the released variety, offering long-term effectiveness of traits, thus allowing breeders to “lock in” favorable genetic combinations, making MAS particularly impactful and durable in these crops.

#### 3.5.2 Trait architecture

Like MAS in any other crop, the success of MAS in RTB crops depends heavily on the genetic architecture of the target trait. MAS has proven most effective for traits controlled by one or a few major genes, such as disease resistance. For example, in cassava, resistance to cassava mosaic disease (CMD) is largely governed by the CMD2 gene, which has enabled reliable early selection using molecular markers. However, while allele-specific MAS is highly effective for major-effect loci, small-effect QTL also play an important role, particularly in complex traits. These minor QTL can be leveraged through a combined approach that integrates allele-specific MAS with genomic selection, enhancing the accuracy and efficiency of breeding programs by capturing both major and polygenic components of trait variation.

#### 3.5.3 Need for rigorous validation

RTB crops are characterized by species and subspecies within the same crop. QTL are often identified in specific species and subspecies. Through validation, the QTL associated with resistance to Foc SR4 and TR4 in bananas and plantains was found valid in the M. acuminata spp. malaccensis genetic background only (Chen et al., 2023b). This finding underscores the critical importance of genetic background in the expression of resistance trait and the need to expand QTL mapping efforts. Each MAS deployment must be preceded by a rigorous validation, and the application must be tailored to the particular genetic background of the target breeding population to ensure effective selection and stable expression of the traits.

#### 3.5.4 From individual SNP to haplotype calling

Individual SNP markers often lose linkage information, underestimate allelic diversity, and reduce power and prediction accuracy. Recent advances in molecular breeding highlight the advantages of haplotype-based marker panels over traditional individual SNP markers (Brinton et al., 2020; Sehgal et al., 2020). Haplotype markers, which capture blocks of linked genetic variation, offer greater predictive stability across diverse populations and can reduce genotype misclassification, a common issue in highly heterozygous and polyploid RTB crops. As vegetatively propagated crops, RTBs are characterized by limited natural recombination, resulting in slow linkage disequilibrium (LD) decay, where large segments of the genome are inherited as intact blocks over generations. Haplotypes, which capture these linked alleles, are therefore more informative than individual SNPs in such crops. Because LD persists over longer genomic distances, haplotypes better reflect the functional genetic variation and improve the accuracy of trait-marker associations and selection in breeding programs. Already adopted in many crops including sweetpotato (Yan et al., 2024), haplotype-based markers are yet to be fully utilized in cassava, yam, and banana/plantain. However, the recent development of haplotype-resolved reference genomes in these crops provides a strong foundation to accelerate their adoption and integration into genomic-aided breeding efforts.

#### 3.5.5 Integrating the available tools

To accelerate genetic gain in modern breeding programs, there is a growing need to integrate available tools, such as genomic selection, marker-assisted selection, and gene editing, into a unified pipeline. As above-mentioned, genomic selection will benefit from QTL analysis by incorporating markers from low-effect QTL, thereby improving predictive accuracy for complex traits. This enhances the overall reliability and efficiency of both MAS and GS strategies, ensuring that only functionally validated alleles are deployed in breeding programs. The strategic use of gene editing in breeding parents, rather than in final products, offers a flexible and powerful approach to support this integration. Editing elite or donor parents enables the precise introduction of desirable alleles early in the breeding process, allowing for the development of multiple progeny that carry the edited trait in combination with other favorable alleles. This approach maximizes the impact of each editing event by influencing a broader range of breeding outputs and facilitating the stacking of multiple traits through conventional recombination. In addition, gene editing serves as a valuable tool for QTL validation. Once QTLs are identified through mapping or genomic studies, candidate genes within these regions can be precisely edited in parent lines to confirm their functional role. This enhances the reliability of MAS and genomic selection strategies by ensuring only functionally validated alleles are deployed in breeding programs.

## 4 Enabling technologies and environments

### 4.1 Improved phenotyping tools and protocols

Precision phenotyping is essential for the development and implementation of genomic tools, as it provides accurate, high-resolution measurements of complex traits using advanced technologies such as imaging, sensors, and automated data collection. By enabling stronger genotype-phenotype associations, it enhances genome-wide association studies, improves genomic selection models, and accelerates the identification and validation of trait-linked markers and candidate genes for crop improvement. RTB breeding teams have invested in improved experimental designs and developing more precise and mid- to high-throughput protocols to improve the quality of phenotypic data (Nakato et al., 2024; Kisitu et al., 2025). The systematic documentation of standard operating procedures for these protocols and other breeding operations ensures data quality, reproducibility, and consistency across research centers (Kanaabi et al., 2021; Agre et al., 2025).

The use of unmanned aerial vehicles (UAVs) and advanced drone systems has been crucial in the high-throughput phenotyping revolution in RTB crops. These drones are revolutionising conventional phenotyping protocols of underground traits. In cassava, for example, research has demonstrated that 3D canopy architecture metrics can accurately predict root expansion patterns, with correlation coefficients ranging from 0.82 to 0.91 (Araus et al., 2018; Nascimento et al., 2025). In the same way, short-range NIRS tools have been implemented to estimate dry matter content by identifying starch-specific spectral signatures in canopy leaves, operating within the wavelength range of 900–1700 nm. In yam, thermal-RGB algorithms have been employed to analyse diurnal temperature fluctuations in vine canopies, thereby reducing the necessity for destructive sampling by 60-75% and facilitating predictions of tuber set and development.

Multi-sensor drone systems have also been deployed to monitor growth dynamics for RTB crops, but at varying levels. For example, in cassava, monthly monitoring of features such as canopy closure rates, leaf area index (LAI) dynamics, and stem elongation patterns during the 3–9 months after planting (MAP) period are being assessed for their relationship with final root yield. Similarly, drone-acquired data has helped researchers analyse yam vine architecture by studying crucial characteristics such as vine climbing angles, internode lengths, and leaf orientation. These traits are essential for increasing light interception and trellising efficiency.

### 4.2 Digital and decision support tools

In RTB, digital tools are playing an increasingly critical role in enhancing the precision, speed, and efficiency of breeding programs. Core to this transformation is the adoption of Breedbase across all RTB crops (cassava, yam, banana, and sweetpotato) as a centralized data platform for managing phenotypic, genotypic, and environmental data (Morales et al., 2022; Arnaud et al., 2023). Breedbase enables comprehensive trait tracking, supports genomic selection and parent selection, and includes a selection index tool for multi-trait decision-making. It has also enabled workflow digitization through barcoding, electronic data capture, and cloud-based integration, thus improving data quality, reduced errors, and enabled real-time analytics.

Complementing Breedbase are specialized global platforms such as the Banana Genome Hub (Droc et al., 2013), the Musa Germplasm Information System (MGIS) (Ruas et al., 2017), and the Crop Ontology (Matteis et al., 2013). These resources provide access to annotated genomes and pangenomes, gene expression data, and passport information that support trait discovery, comparative genomics, and identification of key structural variations relevant to seed set, stress tolerance, and yield-related traits. The Crop Ontology, which is integrated with the Breedbase, ensures harmonized trait definitions and standardized phenotypic protocols across breeding networks.

Additionally, to support data-driven breeding, the CGIAR has developed Bioflow (Delgado et al., 2024), an analytics platform that integrates phenotypic, genotypic, and omics data. Bioflow offers modules for selection index computation, cross-prediction, and genomic-assisted decision-making. This tool is increasingly used across RTB programs and has been integrated into training initiatives to build capacity among CGIAR and NARES partners. Similarly, the Enterprise Breeding System (EBS), recently endorsed by CGIAR, aims to modernize breeding operations and facilitate breeding networks. RTB programs are currently piloting EBS functionalities to assess its suitability for clonally propagated crops. Simulation tools like AlphaSimR (Gaynor et al., 2021) further enable breeders to evaluate breeding scheme design and optimize resource allocation for maximum impact. Collectively, these platforms and tools constitute a robust digital ecosystem that empowers RTB breeding programs to adopt modern, data-driven approaches, accelerating genetic gains and improving breeding outcomes in support of food security and climate resilience.

### 4.3 Induced flowering, increased seed set, and rapid multiplication: additional requirements

Slow production of planting material, long crop cycle, poor flowering, asynchronous flowering, and poor seed set are common breeding challenge that affects RTB crops (Agre et al., 2020; Mondo et al., 2020; Scott, 2021). In cassava, asynchronous flowering is complicated by the fact that 32% of elite genotypes exhibit non-flowering phenotypes, and the female flower receptivity window is critically limited (Rodrmguez et al., 2023). Despite advances in genomic tools, breeding of RTB crops will remain constrained unless physiological challenges related to flowering and multiplication are effectively addressed. A number of solutions to overcome these challenges have proven promising. In cassava, experiments have shown that pruning coupled with the application of anti-ethylene silver thiosulfate increases the number of flowers, while benzyladenine increases the number of female flowers (Hyde et al., 2024; Obare et al., 2024). Furthermore, early flowering has been induced through the extension of the photoperiod using artificial red light-emitting diodes (LED) lighting, reducing the flowering time by 2–3 months (Baguma et al., 2023; Rodrmguez et al., 2023). These techniques are jointly applied in the IITA cassava breeding program to achieve speed breeding, to reduce the breeding cycle, and increase progeny numbers from the intended crosses. In banana and plantain, seed production is extremely low, particularly in landraces, which are nearly infertile due to their triploid nature (2*n* = 3*x* = 33). These landraces have recorded the highest average of only 1.5 seeds per bunch per genotype (Batte et al., 2019). The application of 3% glucose water solution on the banana female flowers before pollination increased up to threefold, while pollinating before anthesis and the use of a saline solution did not give any positive results (Waniale et al., 2021, Waniale et al., 2024). The challenge of the slow multiplication rate of planting materials of RTB crops has also been tackled by advanced multiplication techniques that don’t rely on field production. In cassava and yam, Semi-Autotrophic Hydroponics (SAH) and aeroponics technologies have proven useful to increase the number of cuttings per genotype (Balogun et al., 2014; Binzunga et al., 2024). In banana and plantain, both micropropagation (tissue culture) and macropropagation techniques provide more planting material compared to traditional propagation through field suckers (Hemanthakumar and Preetha, 2023). The plantlets produced through micropropagation are free from pests, fungal and bacterial diseases and, if initiated from virus-free mother plants, can also be free from viral infections. These innovations have facilitated germplasm exchange and early multi-environment testing (MET) across TPEs and significantly supported the RTB seed systems as delivery systems (Legg et al., 2022). Given that novel genomics tools such as GS and MAS have demonstrated greater efficacy during the early testing stages of most RTB crops, the incorporation of rapid multiplication tools holds significant potential for rejecting a substantial number of undesirable genotypes.

## 5 Prospects and recommendations

### 5.1 Integration of emerging trends in breeding: artificial intelligence and machine learning

Artificial intelligence (AI) and machine learning (ML) are increasingly being integrated into breeding pipelines to manage large-scale multi-omics data, improve genomic prediction accuracy, and automate phenotyping. Deep learning models, in particular, are enabling breeders to detect complex, non-linear trait relationships, significantly enhancing selection efficiency (Azodi et al., 2019; Sandhu et al., 2021). AI is also accelerating the development of decision-support systems that assist in ideotype design, environmental risk modelling, and resource optimisation. When combined with environmental covariates, ML models can improve genotype × environment interactions (G×E) predictions and support climate-resilient variety development (Crossa et al., 2017). These tools hold great potential for RTB crops to convert the available large datasets into practical tools to accelerate breeding.

### 5.2 Recommendations for sustained impact

To ensure long-term success and sustainability of genomic-assisted breeding in RTB crops, we propose the following recommendations that reflect both global trends and the specific challenges of these clonally propagated, vegetatively reproduced crops:

1. Strengthen interdisciplinary capacity in RTB-focused breeding teams, particularly by integrating genomics, quantitative genetics, and bioinformatics expertise. Many RTB breeding programs face a chronic shortage of trained personnel capable of applying advanced genomic and AI-driven tools to highly heterozygous, polyploid, or clonal crop species.
2. Invest in open-access genomic platforms tailored to RTB crops, including reference genomes, marker databases, and low-cost genotyping solutions (e.g., skim sequencing, GBS). Such platforms are crucial for scaling up genomic selection and accelerating trait discovery, especially for complex traits like tuber quality, disease resistance, and climate resilience.
3. Establish and strengthen regional RTB genomic innovation hubs, with shared high-performance computing infrastructure, data management pipelines, and multi-environment trial (MET) networks. These hubs should serve as centres of excellence for breeding informatics, phenomics, and genotyping services.
4. Promote participatory and gender-responsive breeding in RTB systems, where end-user preferences, especially for quality traits like taste, texture, storability, and cooking behaviour are central to adoption. This can be facilitated through decentralised variety development, on-farm testing, and feedback loops involving women, youth, and processors.
5. Support agile policy and biosafety frameworks tailored to vegetatively propagated crops, particularly for gene-edited RTB varieties. National regulatory systems often lag in providing clear guidance on genome editing and need to be aligned with regional harmonisation efforts (e.g., AU-NEPAD, ECOWAS).
6. Embed impact-orientated metrics into RTB breeding pipelines, including genetic gain, adoption rates, gender-equity impact, nutrition, and resilience under climate change. These metrics should be used not only for donor accountability but also to guide investment prioritisation and resource allocation across crop programs.

The genetic architecture of RTB crops, along with their critical role in food security, livelihoods, and cultural diets, particularly in Africa, Asia, and Latin America, makes it essential that genomic innovations are both scalable and context-specific. Addressing the bottlenecks in delivery, adoption, and system integration will be vital to ensuring that these technologies translate into tangible improvements for RTB-based food systems.
